# The expression of lnc-CCDC170-4:1, ESR1, lncRNA SRA, and CYP19A1 in cervical squamous cell carcinoma and their relationship with the clinical characteristics

**DOI:** 10.3389/fonc.2024.1430826

**Published:** 2024-08-14

**Authors:** Jinrui Yuan, Mengke Wen, Amina Matnuri, Shihong Zhao, Ning Jian, Guqun Shen

**Affiliations:** The Second Department of Gynecological Surgery, The Affiliated Tumor Hospital of Xinjiang Medical University, Urumqi, China

**Keywords:** lnc-CCDC170-4:1, ESR, lncRNA SRA, CYP19A1, estrogen

## Abstract

**Introduction:**

The occurrence of cervical cancer may be related to estrogen and estrogen receptors. This study investigated the expression of lnc-CCDC170–4:1, *ESR1* (estrogen receptor 1), lncRNA SRA, and *CYP19A1* (aromatase) in cervical squamous cell carcinoma tissues, as well as their relationship with the clinical characteristics of patients.

**Methods:**

Whole transcriptome sequencing analysis was performed on cervical squamous cell carcinoma tissues (n=4) and normal tissues (n=4). The expressions of lnc-CCDC170–4:1, *ESR1*, lncRNA SRA, and *CYP19A1* were validated in 26 cases of cervical cancer tissue and 30 cases of normal cervical tissue using qRT-PCR. The relationship of gene expression with the clinical characteristics and 5-year overall survival rates of cervical cancer patients was analyzed.

**Results:**

The expression levels of CYP19A1 and lncRNA SRA were upregulated, while those of ESR1 and lnc-CCDC170–4:1 were downregulated in cervical squamous cell carcinoma tissue. However, their expression was not related to 5-year overall survival rates (p>0.05). Low expression of lnc-CCDC170–4:1 was associated with lymph node metastasis (p=0.030) and Tumor size (p=0.047), Low expression of ESR was associated with FIGO Staging (p=0.041)and Tumor size(p=0.002),High expression of LncSRA was associated with FIGO Staging(p=0.004).

**Conclusion:**

Estrogen and estrogen receptors may play a role in the occurrence and development of cervical squamous cell carcinoma. Low expression of lnc-CCDC170–4:1 and ESR1 are associated with lymph node metastasis and FIGO stage, so it may be a potential biomarker to evaluate the prognosis of cervical cancer.

## Introduction

Cervical cancer is the fourth-leading cause of cancer-related death in women (1). Despite the rapid development in prevention and treatment strategies over the past few decades, the prognosis for late-stage or recurrent cervical cancer patients remains poor (2). Cervical squamous cell carcinoma exhibits a notably improved prognosis compared to other types of cervical cancer. Patients diagnosed with advanced clinical stage of cervical squamous cell carcinoma present increased rates of lymph node metastasis and reduced 5-year survival rates, which indicate an unfavorable prognosis. Furthermore, the degree of differentiation significantly impacts the postoperative prognosis of cervical squamous cell carcinoma, with 5-year survival rates of 100%, 75%, and 55% observed for high, moderate, and low differentiation, respectively (3). Therefore, investigating the pathogenesis of cervical cancer, identifying prognosis factors, and improving the prognosis is of great clinical significance (4).

It is currently believed that long-term use of oral contraceptives and multiple pregnancies can increase the risk of cervical cancer, indicating that estrogen and estrogen receptors (ER) are closely related to cervical cancer (5, 6). Estrogen receptor inhibitors play an important role in the treatment of breast cancer. Studies have shown that estrone has a pro-inflammatory effect, can increase the expression of embryonic stem cell transcription factors, and induce the epithelial-mesenchymal transition of ER+ HeLa cells (7, 8). *ESR1* (estrogen receptor 1) is the encoding gene for estrogen receptor-α (ER-α) and has been shown to play an important role in mediating the invasion and progression of cervical cancer with ER-α expression loss (9). It has been shown that there are *ESR1* mutations in patients with cervical squamous cell carcinoma (10). However, the prognostic properties of ESR1 in cervical cancer have been less studied.

P450 aromatase is encoded by the *CYP1A1* (aromatase) gene and participates in the hydroxylation metabolism of estradiol. There is a significant correlation between CYP1A1 and the infiltration level of T cells in cervical cancer (11). In addition, the high expression of CYP1A1 and T cells in cervical cancer is unfavorable for the prognosis of cervical cancer patients. Our previous study found that P450 aromatase expression was increased and ER-β expression was decreased in local cervical tissue, indicating that they may play a role in the occurrence and development of cervical squamous cell carcinoma (12).

Long non-coding RNAs (lncRNAs) play an important role in the occurrence and development of tumors (13). Recently, the abnormal regulation of lncRNAs in cervical cancer cells and tissues has been reported 11. Some of these lncRNAs include homeobox transcript antisense RNA, H19, metastasis-associated lung adenocarcinoma transcript 1 (2), cervical carcinoma high-expressed 1, antisense noncoding RNA in the inhibitors of cyclin-dependent kinase 4, growth arrest special 5, and plasmacytoma variant translocation 1. These lncRNAs have been found to play a role in various disease-related processes such as cell growth, cell proliferation, cell survival, metastasis, invasion, and therapeutic resistance (11). The lncRNA SRA facilitates the migration and invasion of cervical cancer cells via the NOTCH signaling pathway and by upregulating MMP-2, MMP-9, and VEGF (14). Additionally, it could potentially function as a therapeutic target and prognostic marker for cervical cancer. Therefore, lncRNAs may serve as potential targets for cancer treatment.

Herein, this study explores the expression of mRNA and corresponding lncRNA related to estrogen and ER in cervical squamous cell carcinoma and their relationship with clinical pathological features (such as tumor size and lymph node metastasis) of patients with cervical squamous cell carcinoma. Our results may provide new ideas for the prognosis assessment of cervical squamous cell carcinoma.

## Materials and methods

### Study participants

Four patients with primary cervical squamous cell carcinoma diagnosed by the Pathology Department of the Affiliated Tumor Hospital of Xinjiang Medical University in October 2017 were enrolled, along with four control individuals who underwent simple hysterectomy due to uterine leiomyoma. The cervical cancer tissues and normal cervical tissues were collected for whole transcriptome sequencing. Additionally, 26 patients with primary cervical cancer diagnosed by the same department from October 2017 to March 2018 and 30 control individuals who underwent simple hysterectomy due to uterine leiomyoma were also recruited. The cervical cancer tissues and normal cervical tissues were collected for quantitative real-time PCR (qRT-PCR) validation. The inclusion criteria for cervical cancer patients included: 1) non-pregnant patients with primary cervical cancer; 2) patients without immunodeficiency disease or other malignant tumors; 3) patients who had no prior history of chemotherapy or pelvic radiotherapy; 4) patients with pathologically confirmed cervical squamous cell carcinoma. The exclusion criteria for cervical cancer patients included: 1) Non-cervical squamous cell carcinoma confirmed by cervical biopsy and pathology at the Affiliated Cancer Hospital of Xinjiang Medical University; 2) Patients with other malignant tumors; 3) After the medical staff explained the purpose of the study, they were unwilling to cooperate Investigation of patients. The inclusion criteria for control individuals included: 1) non-pregnant individuals; 2) individuals without immunodeficiency disease or other malignant tumors; 3) individuals with negative cervical cytology results; 4) individuals with uterine leiomyoma. The exclusion criteria for control individuals included: 1) Abnormal uterine cervical cytology; 2) Those who are unwilling to cooperate with the investigation after the medical staff has explained the purpose of the research to Patient; 3) Patients with other malignant tumors. All procedures and studies involving human subjects to have been carried out according to the ethical guidelines outlined in Declaration of Helsinki. This study was approved by the Ethics Committee of the Affiliated Tumor Hospital of Xinjiang Medical University (No.: K-2021018). All participants have signed the informed consent form.

### Data collection

The basic clinical data were collected, such as patient age, menopausal status, tumor size, lymph node involvement, vascular invasion, FIGO stage, etc.

### Whole transcriptome sequencing

Total RNA was extracted from four pairs of frozen cervical cancer and normal cervical tissues using TRIzol reagent (TransGen Biotech, Beijing, China). The purity and quantity of RNA were assessed using a NanoDrop 2000 spectrophotometer (Thermo Scientific, Waltham, MA, USA). RNA integrity was evaluated using an Agilent 2100 bioanalyzer (Agilent Technologies, Santa Clara, CA, USA). After quality control, the RNA underwent fragmentation, followed by the synthesis of a single-stranded cDNA using six-base random primers. A double-stranded cDNA synthesis was then performed, with the dTTP replaced with dUTP. Different adapters were ligated. The cDNA strand containing dUTP was digested with the Uracil DNA N-Glycosylase. The cDNA single strand with adapters was purified using Agencourt AMPure XP (BECKMAN COULTER, CA, USA) and then underwent end-repair, A-tailing, and ligation of the sequencing adapters. Subsequently, PCR was performed. After purifying the PCR products using AMPure XP beads (BECKMAN COULTER), the library was obtained. An Agilent 2100 chip (Agilent Technologies, SantaClara, CA, USA) was used to detect insert fragments, and the effective concentration was quantified using qRT-PCR to ensure library quality. Finally, the library was sequenced on the Illumina HiSeq X Ten platform (Illumina, San Diego, CA, USA).

### Sequencing data analysis

The quality of sequencing data was typically above Q30, which met the requirements for subsequent analysis. After removing the adapter information and low-quality bases low-quality bases that are present in the original data, clean reads were obtained. We used HISAT2 v2.1.0 to align clean reads with the specified reference genome, and the aligned reads on the specified reference genome were called Mapped Reads. We used Cufflinks v2.2.1 to splice Mapped Reads into transcripts. Using reference transcripts as the library, we used the sequence similarity alignment method of bowtie2 v2.3.1 and eXpress v1.5.1 software to calculate the expression level of each transcript in each sample.

### Identification of differentially expressed genes and gene function analysis

The CuffDiff software was used to identify differentially expressed genes, with the screening criteria of p < 0.05 and a fold change greater than 2. The DAVID tool (https://david.ncifcrf.gov/) was used for Gene Ontology (GO) analysis. KEGG database was used for pathway analysis of differential transcripts.

### Validation with qRT-PCR

Total RNA was extracted using TRIzol reagent (TransGen Biotech) and reversed transcribed into cDNA using 5X All-In-One RT MasterMix (ABM, Shanghai, China). The qRT-PCR reaction mixture comprised 2×SYBR Green Select Mix (5 μL), cDNA (1 μL), upstream primer (0.7 μL), downstream primer (0.7 μL), and enzyme-free water (10 μL). The PCR reaction conditions were as follows: 95°C for 2 min, followed by 40 cycles of 95°C for 5s and 60°C for 30s. The qRT-PCR experiment was conducted in triplicate, and the relative expression level of the gene was determined using the 2-ΔΔct method. The primers are listed in **Table 1**.

**Table 1:** Primer sequences.

Primer name	Sequence (5’ to 3’)
ESR1-F1	CCCACTCAACAGCGTGTCTC
ESR1-R1	CGTCGATTATCTGAATTTGGCCT
Lnc-CCDC170-4-F1	CCCCTGTACCTCAGAACC
Lnc-CCDC170-4-R1	CAATGGAAGCGGTAGAAC
Lnc-SRA-F1	TCTCAGAGGGTTGTAGGGT
Lnc-SRA-R1	CATTCACGTCCGGTAGCC
CYP19A1F1	GTCGCGACTCTAAATTGCCC
CYP19A1R1	CATCCACAGGAATCTGCCGT

### Follow-up

Patients were followed up by telephone or outpatient visits. The follow-up deadline was March 2023, and the overall survival (OS) was calculated from the surgery until the end of the follow-up period or death.

### Statistical analysis

Statistical analysis was performed using SPSS 26.0 software, and all data are represented as mean ± standard deviation. T-test or one-way analysis of variance compared data of normal distribution, while the rank-sum test analyzed data of non-normal distribution. The independent sample t-test was used to evaluate the relationship of lnc-CCDC170–4:1, *ESR*, lncRNA SRA, and *CYP19A1* expression with clinical pathological features. Correlation analysis was conducted with Pearson’s correlation. The Kaplan-Meier method analyzed the OS of patients. P < 0.05 indicates a significant difference.

## Results

### Results of whole transcriptome high-throughput sequencing

**Figure 1:**
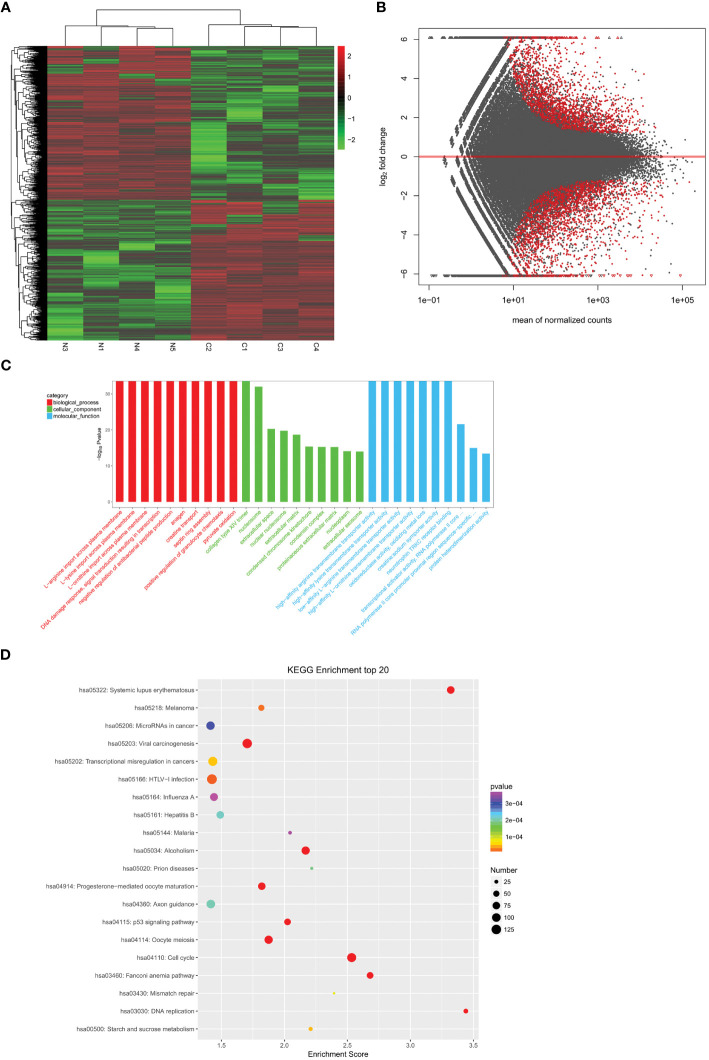
Analysis of differentially expressed genes. **(A)** A heatmap showing differentially expressed mRNAs between cervical cancer tissues (n =4) and normal cervical tissues (n =4). **(B)** The volcano map showing the differential transcripts. **(C)** The bar diagram showing the Top30 enriched GO terms. **(D)** Example of KEGG enrichment corresponding pathway map.

To identify differentially expressed mRNAs and lncRNAs in cervical cancer, we analyzed four cases of cervical cancer tissues and four normal cervical cases through whole transcriptome high-throughput sequencing. The results showed a significant increase in transcripts that were differentially expressed in cervical cancer tissues when compared to normal cervical tissues (P<0.05) (**Figures 1A, B**), including 3035 significantly up-regulated genes and 3319 significantly down-regulated genes. We analyzed the functions of the differentially expressed transcripts through GO (**Figure 1C**) and KEGG (**Figure 1D**) enrichment analysis. We calculated the significance of enrichment for each pathway entry using the hypergeometric distribution test. Finally, we screened mRNA related to estrogen and co-expressed and/or related lncRNAs from lncRNA cis and trans mechanisms, namely CYP19A1, lncRNA SRA, ESR, and lnc-CCDC170–4:1.

### Validation of lnc-CCDC170–4:1, *ESR*, lncRNA SRA, and *CYP19A1* expression with qRT-PCR

**Figure 2:**
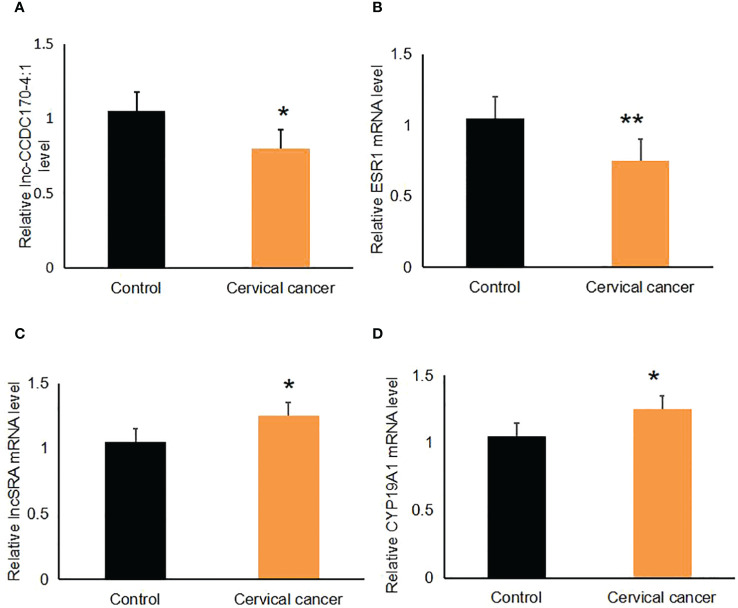
Expression levels of lnc-CCDC170–4:1, *ESR*, lncRNA SRA, and *CYP19A1*. Their levels were measured with qRT-PCR. **(A)** Level of lnc-CCDC170–4:1. **(B)** Level of *ESR*. **(C)** Level of lncRNA SRA. **(D)** Level of *CYP19A1*. *P < 0.05, **P<0.01.

The expression levels of lnc-CCDC170–4:1, ESR, lncRNA SRA, and CYP19A1 were validated using qRT-PCR in 26 cases of cervical cancer tissue and 30 cases of normal cervical tissue. We found that the relative expression levels of lnc-CCDC170–4:1 (**Figure 2A**) and *ESR* mRNA (**Figure 2B**) were significantly lower in cervical cancer tissue than in the control (P<0.05). The relative expression level of lncRNA SRA (**Figure 2C**) and *CYP19A1* mRNA (**Figure 2D**) were significantly higher in cervical cancer tissue.

### Correlation analysis

**Figure 3:**
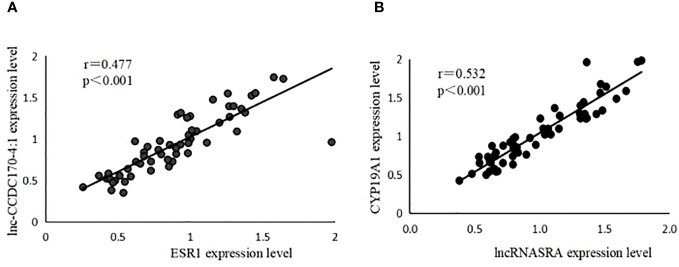
Pearson correlation analysis. **(A)** The positive association between lnc-CCDC170–4:1 and ESR1. **(B)** The positive association between lncRNASRA and CYP19A1.

Pearson’s correlation analysis was used to analyze the correlation between the expression levels of lncRNASRA and *CYP19A1*, as well as lnc-CCDC170–4:1 and*ESR*. The results showed that lnc-CCDC170–4:1 and *ESR* were positively correlated (r=0.477, P<0.001) (**Figure 3A**). Similarly, lncRNASRA and *CYP19A1* were also positively correlated (r=0.532, P<0.001) (**Figure 3B**).

### The effect of lnc-CCDC170–4:1, ESR, and lncRNA SRA on the prognosis of patients

**Figure 4:**
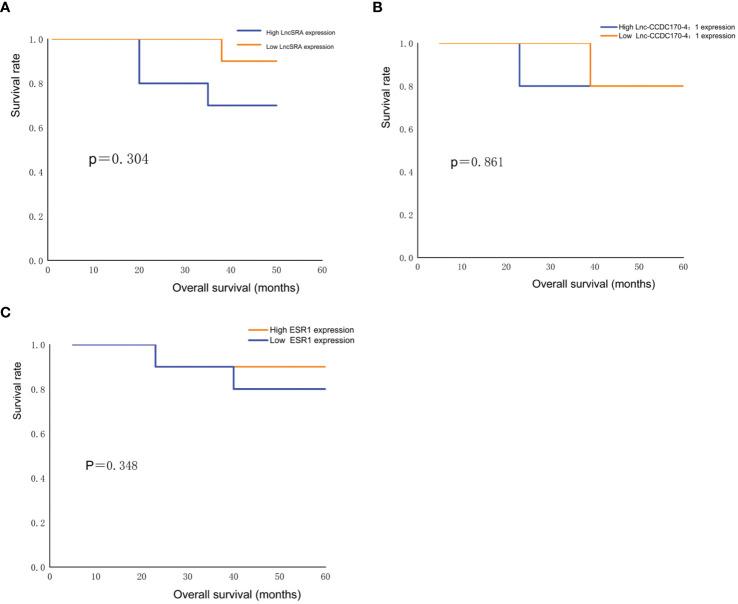
Survival analysis. **(A)** Comparison of survival rate of cervical cancer patients with low and high lncRNASRA expression. **(B)** Comparison of survival rate of cervical cancer patients with low and high lnc-CCDC170–4:1 expression. **(C)** Comparison of survival rate of cervical cancer patients with low and high ESR expression.

Kaplan-Meier survival curve analysis was used to evaluate the OS of 26 cervical cancer patients. The survival rate of cervical cancer patients with high expression levels of lncRNA SRA was lower than that of patients with low expression levels of lncRNA SRA (**Figure 4A**), while the survival rate of cervical cancer patients with low expression levels of lnc-CCDC170–4:1 (**Figure 4B**) and ESR (**Figure 4C**) was lower than that of patients with high expression levels of lnc-CCDC170–4:1 and ESR. However, due to the small sample size, the differences were not statistically significant (P>0.05).

### The association between the expression levels of lncRNA SRA, *CYP19A1*, lnc-CCDC170–4:1, and *ESR* and the clinical-pathological features of patients with cervical cancer

We analyzed the relative expression levels of these lncRNAs and mRNAs in the cancer tissues of 26 patients with cervical cancer and divided the patients into low- and high-expression groups based on the median value. Subsequently, we assessed the relationship between the expression levels of these genes and the clinical-pathological features of cervical cancer patients. The results showed that the expression of lnc-CCDC170–4:1 was associated with lymph node metastasis (p=0.030)and Tumor size(p=0.047), Low expression of ESR was associated with FIGO Staging(p=0.041)and Tumor size(p=0.002) (**Table 2**). High expression of LncSRA was associated with FIGO Staging(p=0.004) (**Table 3**).

**Table 2:** Relationship between Lnc-CCDC170-4:1 and ESR expression and clinicopathological characteristics.

Clinical Features	Lnc-CCDC170-4:1 expression		P-value	ESR expression		P-value
	High	Low		High	Low	
Menopause			0.680			0.680
No	5	4		4	5	
Yes	8	9		9	8	
Tumor size (cm)			0.047*			0.002*
≤4	10	4		11	3	
>4	3	9		2	10	
Lymph node metastasis			0.030*			0.593
Yes	0	4		1	3	
No	13	9		12	10	
Vasculature infiltration			1.000			1.000
Yes	1	2		2	1	
No	12	11		11	12	
FIGO Staging			0.688			0.041*
Ib-IIa	7	9		11	5	
IIb-IIIa	6	4		2	8	

FIGO, International Federation of Gynecology and Obstetrics.

* P < 0.05.

**Table 3:** Relationship between lncRNASRA and CYP19A1 expression and clinicopathological characteristics.

Clinical Features	LncSRA expression		P-value	CYP19A1 expression		P-value
	High	Low		High	Low	
Menopause or not			0.411			1.000
No	6	3		5	4	
Yes	7	10		8	9	
Tumor size (cm)			0.238			0.695
≤4	5	9		6	8	
>4	8	4		7	5	
Lymph node metastasis			0.593			0.593
Yes	3	1		1	3	
No	10	12		12	10	
Vasculature infiltration			0.539			0.539
Yes	1	2		2	1	
No	12	11		11	12	
FIGO Staging			0.004*			0.226
Ib-IIa	12	4		10	6	
IIb-IIIa	1	9		3	7	

FIGO, International Federation of Gynecology and Obstetrics.

* P < 0.05.

## Discussion

Cervical cancer remains one of the main causes of cancer-related death in women worldwide. The malignant proliferation of cervical cancer cells, lymphatic infiltration, and distant organ metastasis are the main reasons for the high mortality rate of cervical cancer patients. More and more studies are focused on prognostic-specific molecular markers in cervical cancer (15, 16). As one of the important female sex hormones, estrogen can regulate gene expression and the function of the female reproductive system, and stimulate the development, proliferation, migration, and survival of target cells by binding with ER (17–19). The cervix is a target organ of estrogen, and most cervical cancers originate from the squamous-columnar junction of the cervix, where the immature metaplastic squamous epithelium is metabolically active and highly hormone-sensitive. Some scholars believe that estrogen may play a carcinogenic role in cervical lesion progression (20). LncRNA in cells regulates the proliferation, invasion, and migration of tumor cells. Thus, lncRNAs related to estrogen and ER may be potential targets for tumor therapy (21).

P450 aromatase is a key enzyme in local estrogen synthesis. Our previous study 12 revealed an increase in P450 aromatase expression in cervical cancer. Due to its involvement in local estrogen production, molecular therapy for breast cancer is based on the inhibition of aromatase to minimize androgen-to-estrogen conversion and attenuate estrogen levels. CYP19A1, a member of the cytochrome P450 superfamily, serves as a monooxygenase during steroidogenesis. It is reported that CYP19A1 was a potential biomarker for gastric cancer prognosis and immune cell infiltration (22). There is evidence that the overexpression of lncRNA SRA can induce EMT, cell proliferation, migration, and invasion *in vitro* (23). As a result, lncRNA SRA may elevate EMT-associated genes, leading to increased cancer cell aggressiveness and making it a promising biomarker for predicting cervical cancer relapse and prognosis as well as a therapeutic target. In this study, we carried out qRT-PCR to evaluate the lncRNA SRA expression and its corresponding mRNA, *CYP19A1*, in cervical cancer and control tissues. We found a significant increase in lncRNA SRA expression in cervical cancer tissues compared to the control, while the expression of *CYP19A1* did not significantly differ between the two groups. Moreover, we found a positive correlation between the lncRNA SRA and *CYP19A1* expression levels, despite the regulatory mechanism still requiring further investigation. Further, they had no significant effect on patient survival. Additionally, we analyzed the relationship between the expression of lncRNA SRA and CYP19A1 and the clinical-pathological characteristics of 26 cervical cancer patients. We found that high expression of LncSRA was associated with FIGO Staging, it may be an important factor for evaluating the prognosis of cervical cancer.

In addition, ESR1 is a nuclear factor that mediates the biological effects of estrogen and is the encoding gene for ERα. Studies (24, 25) have shown that if the ESR1 gene is overexpressed in breast cancer cells, the risk of postmenopausal women developing ER-positive breast cancer increases. Chen et al. (26) found that overexpression of ESR1 partially reversed the effect of miR-944 on the expression levels of E-cadherin, N-cadherin, and Vimentin in cervical cancer cells. Wen et al. (27) revealed that the expression of ERα gradually decreased with the progression of cervical lesions, which may become a diagnostic or staging indicator for cervical cancer. Tumor size and lymph node metastasis are independent survival factors for cervical cancer patients (28). In this study, we investigated the role of ESR1 and its corresponding lnc-CCDC170–4:1 in cervical cancer. Our findings showed that the expression of *ESR1* and lnc-CCDC170–4:1 was downregulated in cervical cancer tissues, and the decreased expression of lnc-CCDC170–4:1 was associated with lymph node metastasis and Tumor size, expression of ESR was associated with FIGO Staging and Tumor size, may be an important target for prognosis evaluation in cervical cancer patients.

It is known that estrogen and ER play an important role in the treatment of breast cancer. If ERα plays an important role in the development of cervical cancer, drugs targeting ER may also play a role in the treatment of cervical cancer (29). It has been shown that ERα can be used as a biomarker to evaluate the OS of HPV-positive oropharyngeal cancer patients (30). Therefore, the lnc-CCDC170–4:1 and ESR1 may be an important factor for evaluating the prognosis of cervical cancer, and their specific mechanism of action in cervical cancer is also one of our main research directions in the future.

## Conclusions

In conclusion, the expression of lncRNA SRA and CYP19A1 is elevated in cervical cancer, while lnc-CCDC170–4:1 and ESR1 expression is decreased in cervical cancer. Moreover, low expression of lnc-CCDC170–4:1 and ESR1 are associated with lymph node metastasis and FIGO stage, so it may be a potential biomarker to evaluate the prognosis of cervical cancer.

## Data Availability

The datasets presented in this study can be found in online repositories. The names of the repository/repositories and accession number(s) can be found in the article/supplementary material.
